# Long-Term Dietary Fish Meal Substitution with the Black Soldier Fly Larval Meal Modifies the Caecal Microbiota and Microbial Pathway in Laying Hens

**DOI:** 10.3390/ani13162629

**Published:** 2023-08-15

**Authors:** Junliang Zhao, Takuma Ban, Hironori Miyawaki, Hirofumi Hirayasu, Akihisa Izumo, Shun-ichiro Iwase, Koji Kasai, Kiyonori Kawasaki

**Affiliations:** 1 Graduate School of Agriculture, Kagawa University, Ikenobe 2393, Miki-cho, Kita-gun, Kagawa 761-0795, Japan; cho.shunryo@kagawa-u.ac.jp (J.Z.); bantaku14@gmail.com (T.B.); skantetu1085@icloud.com (H.M.); 2 Research Institute of Environment, Agriculture and Fisheries, Osaka Prefecture, Shakudo 442, Habikino, Osaka 583-0862, Japan; hirayasuh@mbox.kannousuiken-osaka.or.jp (H.H.); izumoa@mbox.kannousuiken-osaka.or.jp (A.I.); iwases@mbox.kannousuiken-osaka.or.jp (S.-i.I.); kasai@mbox.kannousuiken-osaka.or.jp (K.K.)

**Keywords:** insect meal, liver metabolism, plasma biochemical parameter, short-chain fatty acid, gut microbial pathway

## Abstract

**Simple Summary:**

Research has suggested that long-term feeding black soldier fly larvae (BSFL) meal prepared from leftovers-rearing improves the performance of laying hens. However, the effect of BSFL supplements on the physiological metabolism of laying hens is not well understood. This study suggested that incorporating BSFL meal into the diet of laying hens had minimal effect on blood biochemical parameters, hepatic amino acid and saturated fatty acid contents, intestinal mucosal disaccharidase activity, and intestinal morphology. Nevertheless, it increased the abundance of acetic and propionic acid-producing bacteria, caecal short-chain fatty acids, and modified gut microbial pathways, thus may contribute to poultry performance. The findings of this study may aid further research for an in-depth understanding of the effects of BSFL by elucidating the interactions between microbial metabolites and enterocyte metabolism.

**Abstract:**

Feeding laying hens with black soldier fly larval (BSFL) meal improves their performance. However, the beneficial mechanism of BSFL meals in improving the performance of laying hens remains unclear. This study investigated the effects of the BSFL diet on liver metabolism, gut physiology, and gut microbiota in laying hens. Eighty-seven Julia hens were randomly assigned to three groups based on their diets and fed maize grain-and soybean meal-based diets mixed with either 3% fish meal (control diet), 1.5% fish and 1.5% BSFL meals, or 3% BSFL meal for 52 weeks. No significant differences were observed in biochemical parameters, hepatic amino acid and saturated fatty acid contents, intestinal mucosal disaccharidase activity, and intestinal morphology between BSFL diet-fed and control diet-fed laying hens. However, the BSFL diet significantly increased the abundance of acetic and propionic acid-producing bacteria, caecal short-chain fatty acids, and modified the caecal microbial pathways that are associated with bile acid metabolism. These findings indicate that consuming a diet containing BSFL meal has minimal effects on plasma and liver nutritional metabolism in laying hens; however, it can alter the gut microbiota associated with short-chain fatty acid production as well as the microbial pathways involved in intestinal fat metabolism. In conclusion, this study provides evidence that BSFL can enhance enterocyte metabolism and gut homeostasis in laying hens.

## 1. Introduction

The globally increasing demand for animal-sourced foods has imposed increased pressure on feed resources in animal farming. About 33% of arable land has been used for growing food for livestock production, which only produces 25% of all protein for human consumption [1]. The potential challenge of supplying feedstuffs for livestock production may thus need to be addressed to meet the increasing demand for animal-sourced food consumption. In addition, the production of feed proteins for rearing livestock, such as soybean and fish meals, is limited by available arable land, biologically sustainable fish stock falls [2], and unstable fish catches. Considering the above circumstances, extensive studies have been launched to exploit alternative feed proteins that rear livestock efficiently and require less cropland.

The utilization of edible insects as alternative resources for protein has been explored as a prospective solution to achieve environmentally sustainable global food security. Black soldier flies (BSF; *Hermetia illucens*) have attracted increasing attention due to their effective biomass conversion, low pathogenic risk, and high nutritional value. Moreover, the potential of BSF to reduce pathogen (e.g., *E. coli* and *Salmonella* sp.) and mycotoxin (e.g., Aflatoxin and Ochratoxin) contaminations in waste is evident in previous studies [3]. It can also be processed into feed protein ingredients to prevent the accumulation of anti-nutritional factors in plant-based proteins, such as trypsin inhibitors [4]. Therefore, the pertinency of BSF to food recycling, waste management, and feed processing has been extensively explored. Some evidence suggests that using BSF larval (BSFL) meal in aquaculture feed improves the gut and skin mucosal immune responses in fish, consequently contributing to better growth performance [5,6]. Studies have also explored the potential of BSFL meal as an alternative protein ingredient in pig feed without adverse effects on growth performance and gut morphology [7].

In addition to its applications in aquaculture and swine feed, BSFL meal has been extensively investigated as a possible dietary ingredient for poultry, which naturally tend to consume insects. To date, both partially defatted and full-fat BSFL meals have been identified as substitutes for traditional protein and oil sources in the diets of poultry, such as pigeons, barbary partridges, laying hens, and broilers. The effects of these BSF-incorporated diets can vary depending on the host growth phase and physiologic conditions [8,9]. In addition, a consensus has not been reached regarding the appropriate supplement amount of BSF oil and BSFL meal in poultry feed. The complete substitution of soybean oil with BSF oil in diets shows no effect on the birds’ performance and physiology; however, a significant lauric acid in the meat may concern consumers [10,11]. Incorporating BSFL meal as a supplement at a level of 15% in diets has been observed to negatively affect feed conversion ratio and gut microbiota in birds, while the 10% addition of BSFL meal shows a comparable effect to the control diet. Furthermore, the inclusion of 5% BSFL meal positively impacts either the caecal microbiota or the body weight of the birds [12].

Given the beneficial effects of BSFL-incorporated diets on growth performance and gut microbiota in birds, several studies have insight into the profiles of microbial communities harbored in poultry gut microbiota. *Firmicutes*, *Bacteroidetes*, *Proteobacteria*, and *Actinobacteria* are identified as predominant phyla across the dietary treatment [13]. Additionally, feeding with a low level of BSFL (10%) to layers leads to an increase in caecal microbial diversities [14]. Ndotono et al. conclude a similar finding that the gut microbial diversity of laying hens fed with BSFL meal as a complete replacement of fishmeal (10%) is higher than that of laying hens fed with control diets [15]. Furthermore, Biasato et al. indicated that a 5% level of BSFL meal inclusion is more beneficial to the gut health of broilers than the addition of 10% and 15% BSFL meal in terms of changes in gut microorganisms and intestinal mucin production [16].

Diet triggered the alternations in gut microbiota abundance and the host-gut microbiota interplay can intricately affect their performance and physiology. The relative abundance and metabolism of specific microorganisms in gut microbiota, such as *Lactobacillus vaginalis*, *Sporobacter termitidis*, and *Shigella sonnei*, are closely linked to bird health and performance, including body weight [17]. In addition, the changes in amino acid and fatty acid metabolic profiles of gut microorganisms, specifically concerning the digestion of protein and fat present in the BSFL meal, may also serve to impact host performance and metabolism, such as body weight and nutritional composition of laid eggs [12,18]. In a previous study, we observed that BSFL meal could be a potential alternative to fish meal in laying hen diets, resulting in higher body and egg weights [19]. However, our current understanding of how BSFL affects gut microbial metabolism in laying hens is limited. Based on these findings, we hypothesized that incorporating the BSFL meal into poultry diets may affect the interplay between host and gut microbiota. To test this hypothesis, we aimed to investigate the potential effects of a BSFL diet on the liver nutrient metabolism and gut physiology of laying hens.

## 2. Materials and Methods

### 2.1. Experimental Diets, Animals, and Sampling

The animal experiments were conducted with the approval of the Kagawa University Animal Experiments and Ethics Committee (approval code: 19666). Julia laying hens (*n* = 87; 171 days old) with a laying rate of ≥80% were acquired from a commercial farm (Niinobe Hatchery, Kagawa, Japan). The hens were randomly divided into three groups (*n* = 29 in each group) and fed with maize grain and soybean meal-based diets mixed with either 3% fish meal (Yoshida Feed Co., Ltd., Kagawa, Japan; control diet), 1.5% fish meal and 1.5% BSFL meal (L diet), or 3% BSFL meal (H diet) for 52 weeks. The BSFL meal was obtained by heat-killing, drying, pulverizing, and defatting the BSFL reared on a substrate designed to imitate leftovers (cabbage:rice:chicken meat = 65:20:15). The diet composition is presented in Table S1 [19]. All of the laying hens were raised separately in cages with ad libitum access to water and feed. The laying hens were raised in ambient temperature (13 °C to 32 °C), humidity (55% to 85%), and a light and dark cycle of 12 h each.

Samples for this study, including blood, liver, small intestine, and caecal contents, were collected after decapitation and dissection of the laying hens in each group. After the feeding trials, the average body weight of laying hens fed with control diet is 1455.79 g, whereas the average body weight of hens fed with L diet is 1545.03 g, and these fed with H diet is 1573.86 g. In addition, the average weight of eggs from control diet-fed layers is 56.59 g, and that from L and H diets-fed layers are 58.20 g and 59.16 g. The other performance parameters, including feed intake, laying rate, and mortality are presented in Table S2 [19].

### 2.2. Quantification of Free Amino Acid in Liver

The collected liver was lyophilized and then deproteinized with 0.4 M perchloric acid, followed by chloroform extraction to remove fat. After centrifugation (15,000× *g*, 5 min, 4 °C), the obtained supernatant was filtered through a 0.45 μm membrane filter (RephiLe Bioscience, Ltd., Boston, MA, USA) and used as the sample. Total amino acid content was analyzed using a Waters Acquity TQD Liquid Chromatography with tandem mass spectrometry (LC-MS-MS) System (Waters Corporation, Milford, MA, USA) fitted with an Intrada Amino Acid column (100 × 3 mm, 3 μm particle size; Imtakt, Kyoto, Japan). The separation was carried out with an electrospray ionization mode. The mobile phase comprised solvent A (acetonitrile:tetrahydrofuran:25 mM ammonium formate:formic acid = 9:75:16:0.3) and solvent B (20% acetonitrile in 100 mM ammonium formate solution). Gradient elution was performed as follows: 0% solvent B (0 to 3 min), 0% to 17% solvent B (3 to 9.5 min), and 17% to 100% solvent B (9.5 to 16 min). The flow rate was 0.6 mL/min. The parameters used in mass spectrometry are as follows: capillary voltage 2500 V, desolvation gas 1200 L/h, desolvation temperature 550 °C, ion source heater 150 °C, and cone gas 50 L/h.

### 2.3. Quantification of Fatty Acid Concentration in Liver

The fatty acid concentration in the liver was analyzed using a GCMS-QP2010 SE system (Shimadzu, Kyoto, Japan) equipped with an Agilent DB-5MS column (30 m × 0.25 mm, 0.25 μm; Agilent Technologies, Santa Clara, CA, USA). Briefly, ~100 mg of the liver was saponified with 2 mL NaOH (0.5 mol/L) in methanol. The liberated fatty acids were methyl esterified with methanol in 0.5 mol/L boron trifluoride. Methyl-esterified fatty acids obtained from the hexane extraction were used for analysis. The injection was performed in splitless mode. The oven temperature was set to 40 °C for 2 min and then increased to 320 °C at a rate of 6 °C/min and held for 1.07 min. The vaporization chamber, interface, and ion source temperatures were set at 280 °C, 280 °C, and 200 °C, respectively. Data were collected in the scan range of 45 to 500 *m*/*z*.

### 2.4. Measurement of Plasma Biochemical Parameter

Plasma biochemical parameters, including alkaline phosphatase (ALP), glutamic oxaloacetic transaminase (GOT), gamma glutamine transpeptidase (GGT), lactate dehydrogenase (LDH), high-density lipoprotein cholesterol (HDL-c), total cholesterol (T-cho) and minerals, including calcium (Ca), magnesium (Mg), and inorganic phosphorus (IP) were assayed using an automatic biochemical analyzer (EZ SP-4430; ARKRAY, Kyoto, Japan). The immunoglobulin G (IgG) and tumor necrosis factor-alpha (TNF-α) in plasma were examined using Chicken IgG ELISA Kit (LABISKOMA, Seoul, Republic of Korea) and TNF-α ELISA Kit (CLOUD-CLONE CORP, Houston, TX, USA), respectively. All procedures were performed according to the manufacturer’s instructions.

### 2.5. Disaccharidase Activity Test in Intestinal Mucosa

Jejunal and ileal mucosal tissues (50 mg) were homogenized by grinding in PBS buffer with a pestle. The homogenized mucosal tissue was centrifuged (10,000× *g*, 4 min, 4 °C), and the supernatant was collected for disaccharidase activity assay. Maltase and sucrase activities were determined using the QuantiChrom™ α-Glucosidase (BioAssay Systems, Hayward, CA, USA) and Invertase Activity (BioVision Inc., Waltham, MA, USA) Assay Kits, respectively, following the manufacturer’s instructions.

### 2.6. Intestinal Morphological Analysis

Intestinal histological analysis was conducted as described by Kawasaki et al. [14] with a minor modification. The intestinal tissues were fixed using 10% formalin solution for 24 h, followed by dehydration and embedding in ethanol and paraffin, respectively. The intestinal tissue was stained with alcian blue before measuring the villi height, crypt depth, villi-to-crypt ratio, and goblet cell density. The intestinal tissue was observed and analyzed using an optical microscope (BZ-9000; KEYENCE, Osaka, Japan) at 10× magnification and ImageJ software (version 1.54f) [20].

### 2.7. 16S rRNA Gene Amplicon Sequencing and Taxonomic Analysis

DNA was extracted from the caecal contents using a QuickGene DNA tissue kit (DT-S; Kurabo Industries Ltd., Osaka, Japan) following the manufacturer’s instructions. A prokaryotic universal primer, 341F-805R, was used to amplify the V3-V4 region of bacterial 16S rRNA genes [21]. The Next-Generation Sequencing library preparation and sequencing were performed according to the Illumina protocol. All sequencing FASTQ files obtained in this study were deposited in the NCBI Sequence Read Archive, and the BioProject accession number is PRJNA974681. The taxonomic analysis of the obtained data was conducted using the RDP classifier (version 2.13).

### 2.8. Determination of Short-Chain Fatty Acid in Caecal Content

The concentration of short-chain fatty acids (SCFAs) in the caecal content was determined using the GCMS-QP2010 SE system equipped with the Agilent DB-5MS column following the protocol described by Kawasaki et al. [22].

### 2.9. Prediction of Microbial Functional Abundance and Metabolic Pathway Analysis

The functional abundance of the microbial communities was predicted using a phylogenetic investigation of communities by reconstructing unobserved states [23]. Microbial functional profiles were annotated using the Kyoto Encyclopedia of Genes and Genomes (KEGG) database [24]. The obtained KEGG orthologs were subjected to principal component analysis (PCA) and KEGG pathway annotations. The KEGG pathway enrichment ratio was calculated as the number of genes expressed in individuals to the total number of genes in the pathway.

### 2.10. Statistical Analysis

Free amino acids, fatty acids, plasma biochemicals, disaccharidase activities, villus height, crypt depth, goblet cell density, and annotated microbial pathways were analyzed using Jeffreys’s Amazing Statistics Program (Version 0.16, JASP Team, 2022). All data were analyzed using the Shapiro–Wilk and Levene’s tests for normal distribution and homogeneity of variance. The Kruskal–Wallis test with Dunn’s multiple comparison test was conducted to examine the significance of data violating the normal distribution and variance homogeneity tests. One-way analysis of variance (ANOVA) with Tukey’s Honest Significant Difference post-hoc test was used to examine significance when data were subjected to normal distribution and variance homogeneity tests. Alpha diversity of the intestinal microbiota was analyzed using the Kruskal–Wallis test, and beta diversity was assessed using PERMANOVA test in QIIME2. The relative caecal microbial abundance in the genus level was examined using the STAMP software (version 2.1.3) with Welch’s *t*-test. PCA of the KEGG pathways in each group was conducted using the STAMP software (version 2.1.3) [25]. A *p*-value less than 0.05 was considered statistically significant.

## 3. Results

### 3.1. Free Amino Acids in the Liver

Free amino acids in the hen livers are shown in Table 1. The most abundant free amino acids are histidine, serine, lysine, methionine, and glutamate. The concentration of aspartate in the liver of the H diet group was significantly higher than the control and the L diet groups. However, no significant differences were found for the other free amino acids among the different diet-fed hens.

### 3.2. Fatty Acid Concentration in the Liver

The concentrations of four fatty acids, palmitic acid (C16:0), stearic acid (C18:0), linoleic acid (C22:2(n-6)), and oleic acid (C22:0), were quantified as shown in Table 2. No significant difference in the contents of these fatty acids was observed between the control and BSFL diet-fed groups.

### 3.3. Plasma Biochemical Parameters

The plasma biochemical parameters, including ALP, GOT, GGT, LDH, HDL-c, T-cho, Ca, Mg, IP, IgG, and TNF-α, are shown in Table 3. All plasma parameters of laying hens fed different diets were comparable.

### 3.4. Disaccharidase Activities in Intestinal Mucosa

Maltase and sucrase activities in jejunal and ileal mucosae are shown in Table 4. Maltase and sucrase activities were higher in the jejunal mucosa than those in the ileal mucosa. The maltase activity of ileal mucosa in the H diet-fed group was significantly higher than that in the L diet-fed group. However, no significant difference in disaccharidase activity in the jejunal mucosa was observed between BSFL and control diet-fed groups.

### 3.5. Intestinal Morphology

**Figure 1:**
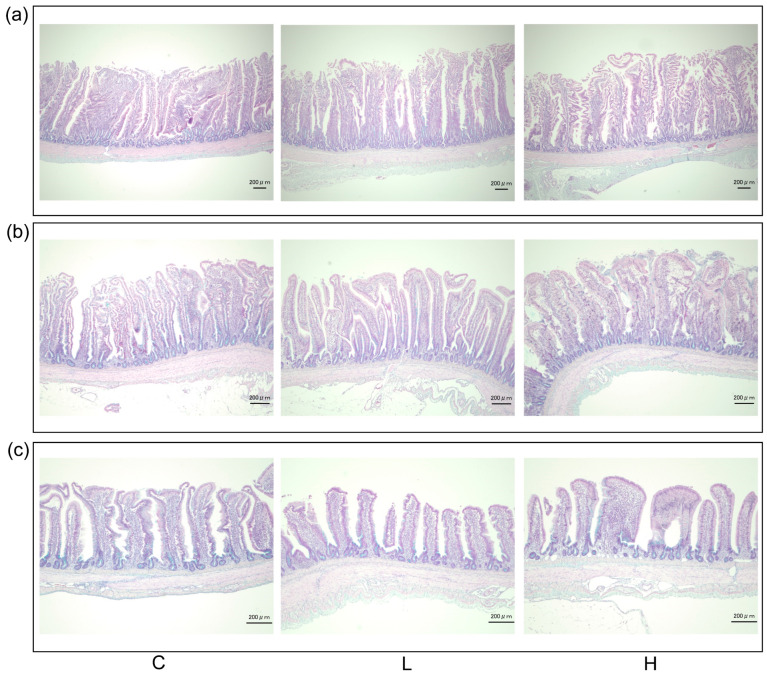
The graphical representation of intestinal morphology in laying hens with different dietary treatments. (**a**) Duodenum; (**b**) Jejunum; (**c**) Ileum. C diet: 3.0% fish meal and 0% black soldier fly larvae (BSFL) meal; L diet: 1.5% fish meal and 1.5% BSFL meal; H diet: 0% fish meal and 3.0% BSFL meal.

The graphical representation of intestinal morphology stained with alcian blue is shown in Figure 1. The villi height, crypt depth, villi-to-crypt ratio, and goblet cell density of the duodenum, jejunum, and ileum are shown in Table 5. No significant differences were observed in these parameters among the different diet-fed groups (*p* > 0.05).

### 3.6. Caecal Microbiota

**Figure 2:**
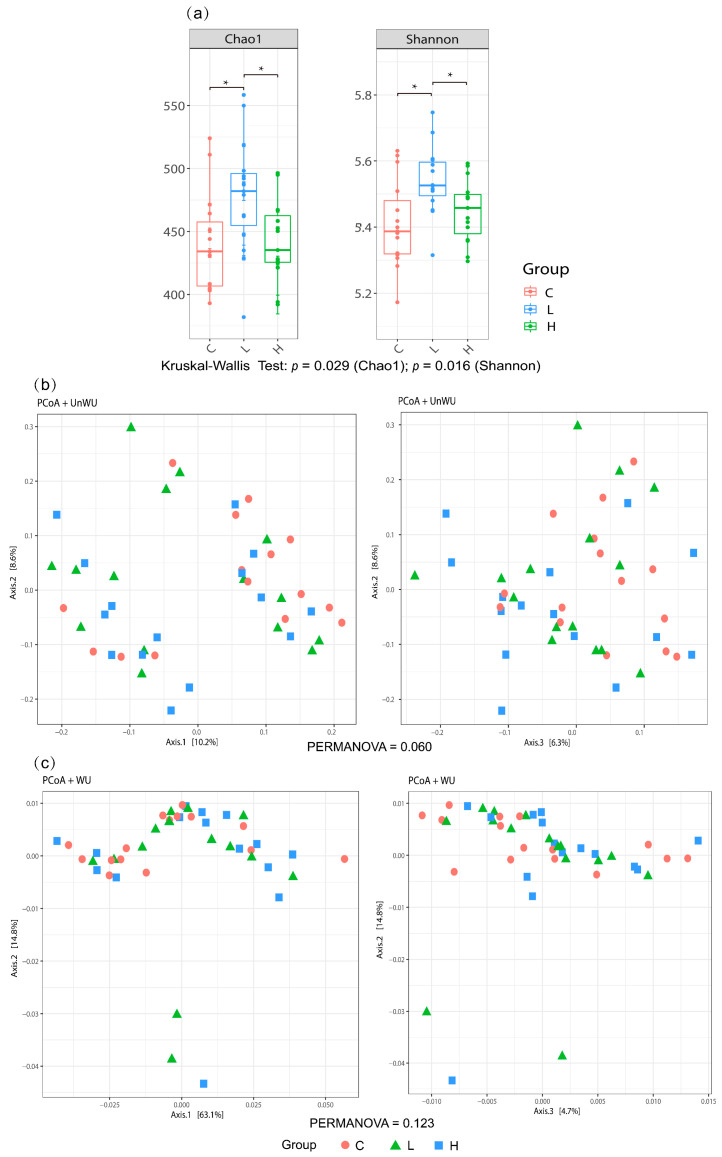
Diversity indices of microbial communities in the gut of laying hens with different dietary treatments. (**a**) α−diversity; (**b**,**c**) principal coordinate analysis plots of (**b**) unweighted UniFrac distance; (**c**) weighted UniFrac distance. C diet: 3.0% fish meal and 0% black soldier fly larvae (BSFL) meal; L diet: 1.5% fish meal and 1.5% BSFL meal; H diet: 0% fish meal and 3.0% BSFL meal. * *p* < 0.05 estimated by pairwise comparison of the α−diversity using Kruskal–Wallis test. Pairwise comparison of the β−diversity was performed using the PERMANOVA test (*p* > 0.05).

A total of 1,273,946 sequences from the caecal contents were generated using the MiSeq system (Illumina, Inc., San Diego, CA, USA). Operational taxonomic unit (OTU) clustering of the sequences identified 2531 OTUs. The α diversity analysis showed that the L diet group had higher Chao 1 (richness) and Shannon indices (evenness) than the control and the H diet groups (*p* < 0.05, Figure 2a). In contrast, β diversity (unweighted UniFrac and weighted UniFrac distance) did not differ significantly among groups (Figure 2b,c).

**Figure 3:**
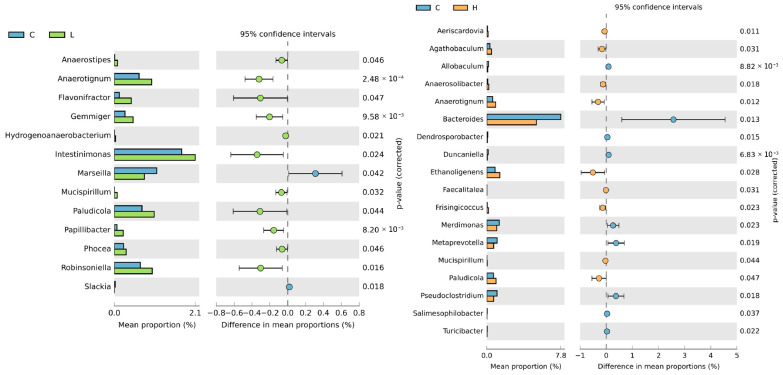
Abundance of caecal microbes at genus level in laying hens with different dietary treatments. C diet: 3.0% fish meal and 0% black soldier fly larvae (BSFL) meal; L diet: 1.5% fish meal and 1.5% BSFL meal; H diet: 0% fish meal and 3.0% BSFL meal.

As shown in Supplementary Figure S1, *Ruminococcaceae*, *Bacteroidaceae*, *Lachnospiraceae*, *Tannerellaceae*, and *Prevotellaceae* were the five most abundant taxa at the family level in the caecal microbiota. In addition, the composition of caecal microbiota differed significantly at the genus level among the different diet-fed groups—the abundance of 13 genera differed between the control and the L diet groups, and that of 18 genera differed between the control and the H diet groups (Figure 3).

### 3.7. SCFAs Concentrations in Caecal Content

**Figure 4:**
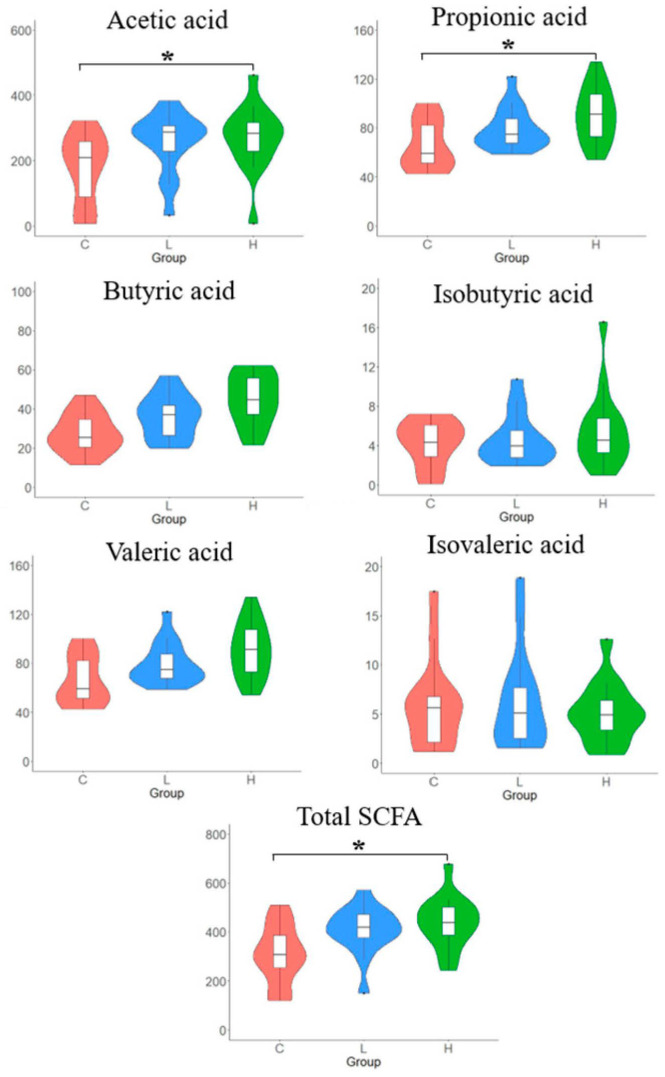
Short−chain fatty acids (SCFAs) in the caecal content of laying hens with different dietary treatments. C diet: 3.0% fish meal and 0% black soldier fly larvae (BSFL) meal; L diet: 1.5% fish meal and 1.5% BSFL meal; H diet: 0% fish meal and 3.0% BSFL meal. * The statistical differences in multiple comparison (*p* < 0.05).

The concentrations of SCFAs in caecal samples are shown in Figure 4. Acetic and propionic acids were the dominant SCFAs in the caecal contents. In addition, the H diet group showed significantly higher concentrations of acetic acid, propionic acid, and total SCFAs than the control group. However, the SCFA content in the L diet group was comparable to that in the control group.

### 3.8. Microbial Functional Abundance Prediction and Metabolic Pathway

**Figure 5:**
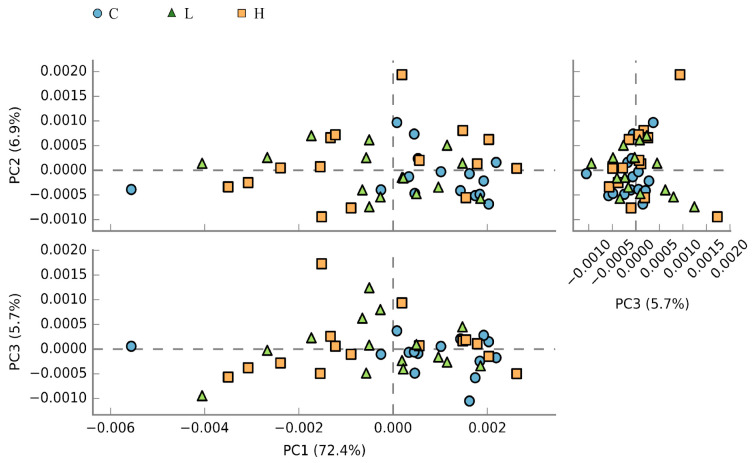
Principal component analysis of the caecal microbial pathways in laying hens with different dietary treatments. C diet: 3.0% fish meal and 0% black soldier fly larvae (BSFL) meal; L diet: 1.5% fish meal and 1.5% BSFL meal; H diet: 0% fish meal and 3.0% BSFL meal.

A total of 10,533 KEGG orthologs (Table S3) were identified and used for PCA analysis and KEGG pathway annotation. The PCA showed that the principal components explained 85.0% of the total variance. Moreover, the transformed data showed differences between the control and BSFL diet-fed groups—the data in the control diet group mainly clustered in the right quadrant of the plot, wherein those in the BSFL diet group clustered in the left and right quadrants (Figure 5). The annotation of the obtained KEGG orthologs resulted in the identification of approximately 6175 microbial pathways. Among the microbial pathways with significant difference, the most abundant ten in the caecal microbiota of laying hens are shown in Table 6. The pathways presented are potentially involved in energy metabolism (e.g., β-glucosidase and α-D-xylosidic diolase) and lipid metabolism (e.g., long-chain acyl-CoA synthetase and bile acid:Na+ synthase). The enrichment ratios of these pathways were higher in the caecal microbiota of laying hens fed with the control diet when compared to those fed with BSFL meal diets. Additionally, a statistically significant difference was observed between the control diet and the H diet groups.

## 4. Discussion

BSFL is widely used to process organic waste into feed ingredients that are rich in protein and fat. The BSFL meal prepared from the leftovers-rearing pattern in the present study contained approximately 52.6% crude protein and 15.0% crude fat, which are considered to potentially affect the amino acid and fatty acid metabolism in the liver of laying hens. However, except for aspartic acid, most free amino acids in the livers of laying hens fed with different diets did not show significant differences. The difference in aspartate could be attributable to the fact that the additional energy requirement by the hens in the H diet group with a higher body was met via the malate-aspartate shuttle [26]. Studies have shown that high-content medium-chain fatty acids, such as lauric acid, in BSFL meals can be rapidly oxidized with low deposition [27]. In the present study, we showed that the fat content of the BSFL meal did not affect lipid accumulation in the liver. No significant differences were observed in saturated fatty acids in the liver, which is consistent with the finding that BSFL meal or extracted fat did not induce liver weight gain in broilers [12,28].

Chitin in BSFL meal is a non-digestible fiber that may negatively influence liver metabolism and function. Previous studies have indicated that replacing fish meals with defatted BSFL meals in aquaculture feed induces liver inflammation in fish [29]. In the present study, the BSFL diet showed no adverse effects on liver function-related enzymes (ALP, GGT, GOT, and GGT), which is concordant with the finding that a defatted BSFL diet did not induce liver damage in Muscovy ducks [30]. In addition, although the BSFL meal used as the fish meal substitute contained approximately 6.5% crude ash, which was less than the crude ash in the fish meal (19.1%), it did not affect the serum mineral metabolism in terms of Ca, Mg, and IP. This result may be contextualized by the findings of other studies [31,32,33]. The non-significant differences in serum HDL-c and T-cho levels supported the finding that the BSFL diet did not affect hepatic lipid metabolism. In addition, comparable serum IgG and TNF-α levels indicated that the BSFL diet did not induce inflammation.

Studies have indicated that BSFL meal used as a soybean meal alternative in poultry feed can modify enzymatic activities in laying hens [34]. The current study demonstrated significantly increased ileal maltase activity in the 3% BFSL meal diet group than that in the 1.5% BFSL meal diet group; however, the change was not significant compared to that in the control diet group. This result contradicts the finding that the BSFL meal substitution for soybean meal in the diet modifies the ileal maltase activity [35], which could be attributed to the different amounts of BSFL meal supplements and various nutritional compositions of the BSFL meals. Nevertheless, the effects of BSFL on intestinal disaccharidase activity require further investigation.

The intestinal villi and crypts are unique structures that contribute to the digestibility and immune response of the host. Replacing fish meal with BSFL meal did not affect the intestinal traits, such as villus height, crypt depth, or villi-to-crypt ratio in the duodenum, jejunum, and ileum. Similar findings have been reported in a previous study that substituted soybean meal in poultry feed with BSFL meal [14]. Bovera et al. [36] indicated that the amount of chitin in insect meals could contribute to the intestinal development of poultry by affecting protein digestibility and, therefore, reducing dry matter digestion. In this study, fat and chitin from the BSFL meal were removed partially; therefore, we speculate that they may contribute to the intestinal development of the laying hens. In addition, the incorporation of BSFL meal into diet did not affect goblet cell density in laying hens, which is consistent with previous findings that a 5% dietary BSFL diet had no effect on mucins staining density [16].

Numerous studies have investigated the effect of BSFL meals on gut microbiota; however, the results are inconsistent. This variation may be ascribed to the diverse composition of the BSFL meal, which is affected by the rearing processes. In this study, the diversity in terms of Chao1 and Shannon indices in the L diet (1.5% BSFL) group was significantly higher than that in the control diet group. In contrast, with increased content of BSFL meal (3.0%) in poultry feed, the α-diversity was reduced to that in the control diet. This result suggests that the complete substitution of fish meal with BSFL meal reduces the diversity of nutritional substances for bacterial growth in the gut, thus affecting the development of various bacteria within individuals. No significant differences were found in β-diversity with respect to unweighted and weighted UniFrac distances. Nevertheless, a significant difference was observed in the relative abundance of some bacteria at the genus level between the different diet groups. The BSFL diet significantly increased the abundance of *Anaerotignum* in the cecum, which produces acetic and propionic acids [37]. In addition, the abundance of some other acetic acid producers, including *Intestinimonas* [38] in the L diet group and *Ethanoligenens* [39] in the H diet group, was also significantly higher than that in the control diet group. The SCFAs produced by the microorganisms collectively confirmed that the BSFL diet significantly increased caecal acetic and propionic acid contents. The abundance of *Paludicola*, which uses chitin as the only growth carbon substrate, was significantly higher in the BSFL diet group, which could be attributable to the digestion of chitin in insect meals [40]. The abundance of *Bacteroides* was higher in the caecal microbiota of laying hens fed the control diet than that in those fed the BSFL diet. However, this did not result in a high acetic acid content, possibly due to the decreased acetic acid production attributed to the differences in carbon sources between the BSFL and fish meals. Additionally, bacteria associated with disease development, such as *Bacteroides* and *Mucispirillium*, were significantly more abundant in the caecal microbiota of hens fed the BSFL diet than in those fed the control diet, but no pathological evidence was observed.

Changes in gut microbial metabolic pathways initiated by the degraded substrates from ingested nutrients can intricately affect gut physiology. The previous study suggested that long-term dietary substitution of BSFL meal for fishmeal increased body and egg weights in laying hens [18], which may attribute to the interplay between the host and gut microbiota. In the present study, although the liver metabolism, intestinal mucosal disaccharidase activity, and intestinal morphology were comparable among the experimental groups, the caecal microbiota abundance was significantly different between the control diet-fed and BSFL meal-fed groups. Indeed, studies have indicated that the caecal microbiota, such as *Parabacteroides*, *Parasutterella*, *Oscillibacter*, and *Anaerofustis*, are involved in fat metabolism and are associated with body weight [41]; however, there was no significant difference regarding these microorganisms in this study. Notably, diets incorporating BSFL meals modified the caecal microbial pathways associated with bile acid metabolism in laying hens, as evidenced by the PCA and microbial pathway results. Studies have suggested that 95% of conjugated bile acids are involved in emulsifying dietary fat and subsequently being reabsorbed from the intestinal epithelium, with the remaining passed through the intestinal tract and excreted via feces. In this study, the caecal microbial metabolism associated with bile acid was lower in the BSFL group compared to the control group. This decrease may be attributed to the high level of bile acids involved in emulsifying the fat from partially defatted BSFL meal in the small intestine, resulting in decreased bile acid excreted into the caecum. This speculation was evidenced by the fact that the *Bacteroides* abundance in the cecum of the control group was significantly higher than that of the BSFL-fed group, as *Bacteroides* display bile salt hydrolase capacity responded for deconjugating the toxic conjugated bile acid [42]. In addition, inhibition of microbial deconjugation of the conjugated bile acid aids in preventing the pathologic development triggered by intestinal permeability, thus protecting the intestinal epithelial barrier [43]. Since diets incorporating BSFL meals have been shown to modify pathways associated with bile acid metabolism, it could conceivably be hypothesized that such a diet may benefit enterocyte lipid digestion and gut physiology. These benefits are in accordance with previous studies showing that a BSFL diet can broadly improve gut health in chickens and fish [15,44]; however, a few attempts have been made to elucidate the relation between the profiles of microbial metabolites and gut physiology. Therefore, the interactions among microbial metabolites, enterocyte metabolism, and gut homeostasis should be investigated for an in-depth understanding of the dietary benefits of BSFL meals.

## 5. Conclusions

Overall, this study suggests that although the BSFL diet had minimal effects on plasma biochemical parameters, hepatic amino acid and saturated fatty acid contents, intestinal mucosal disaccharidase activities, and intestinal morphology in laying hens, it can increase the abundance of acetic and propionic acid-producing microorganisms, the concentration of SCFAs in the cecum, and modify caecal microbial pathways associated with bile acid metabolism, which may contribute to host performance and benefit enterocyte lipid digestion and gut homeostasis in laying hens.

## Figures and Tables

**Table 1:** Free amino acids in the liver of laying hens with different dietary treatments.

Amino Acids (mmol/L)	Diets			*p*-Value
	C Diet	L Diet	H Diet	
Alanine	1.694 ± 0.076	1.648 ± 0.084	1.865 ± 0.133	0.527
Arginine	2.223 ± 0.130	2.045 ± 0.085	2.724 ± 0.238	0.123
Asparagine	0.363 ± 0.029	0.342 ± 0.031	0.491 ± 0.053	0.059
Aspartate	1.749 ± 0.105 ^bc^	1.610 ± 0.080 ^c^	2.275 ± 0.176 ^a^	0.023
Glutamate	2.486 ± 0.143	2.293 ± 0.128	2.868 ± 0.237	0.145
Glutamine	2.752 ± 0.183	2.543 ± 0.149	3.317 ± 0.316	0.181
Glycine	0.757 ± 0.039	0.760 ± 0.041	0.922 ± 0.072	0.171
Histidine	5.480 ± 0.274	5.178 ± 0.270	5.809 ± 0.491	0.783
Isoleucine	1.139 ± 0.068	1.066 ± 0.046	1.435 ± 0.200	0.070
Leucine	1.250 ± 0.073	1.179 ± 0.049	1.582 ± 0.132	0.072
Lysine	3.126 ± 0.184	3.103 ± 0.152	4.090 ± 0.365	0.145
Methionine	2.684 ± 0.146	2.495 ± 0.109	3.378 ± 0.278	0.052
Phenylalanine	1.348 ± 0.077	1.229 ± 0.058	1.436 ± 0.112	0.450
Proline	2.399 ± 0.130	2.287 ± 0.105	2.865 ± 0.245	0.333
Serine	5.326 ± 0.232	4.754 ± 0.221	5.539 ± 0.377	0.611
Threonine	1.473 ± 0.082	1.341 ± 0.062	1.785 ± 0.146	0.065
Tryptophan	0.192 ± 0.010	0.180 ± 0.009	0.191 ± 0.012	0.950
Tyrosine	1.482 ± 0.069	1.350 ± 0.063	1.487 ± 0.117	0.418
Valine	2.504 ± 0.137	2.250 ± 0.102	2.598 ± 0.219	0.458

C diet: 3.0% fish meal and 0% black soldier fly larvae (BSFL) meal; L diet: 1.5% fish meal and 1.5% BSFL meal; H diet: 0% fish meal and 3.0% BSFL meal; Data: mean ± SE; *n* = 15; Mean values within a row with different superscripts represent the statistical differences in multiple comparison (*p* < 0.05).

**Table 2:** Fatty acids in the liver of hens with different dietary treatments.

Fatty Acids (mg/g)	Diets			*p*-Value
	C Diet	L Diet	H Diet	
Linoleic acid (C22: 2(n-6))	0.80 ± 0.16	0.76 ± 0.09	0.84 ± 0.15	0.826
Oleic acid (C22: 0)	5.01 ± 0.73	6.27 ± 1.07	5.85 ± 1.02	0.665
Palmitic acid (C16: 0)	1.32 ± 0.21	1.84 ± 0.45	1.54 ± 0.26	0.693
Stearic acid (C18: 0)	0.77 ± 0.17	0.92 ± 0.18	0.83 ± 0.13	0.377

C diet: 3.0% fish meal and 0% black soldier fly larvae (BSFL) meal; L diet: 1.5% fish meal and 1.5% BSFL meal; H diet: 0% fish meal and 3.0% BSFL meal; Data: mean ± SE; *n* = 15.

**Table 3:** Plasma biochemical parameters of laying hens with different dietary treatments.

Parameters	Diets			*p*-Value
	C Diet	L Diet	H Diet	
ALP (IU/L)	322.86 ± 42.82	365.23 ± 59.12	281.73 ± 25.27	0.806
GOT (IU/L)	112.14 ± 10.86	144.62 ± 11.71	123.20 ± 9.88	0.069
GGT (IU/L)	17.86 ± 1.33	19.38 ± 1.75	18.80 ± 1.26	0.762
LDH (IU/L)	214.86 ± 20.76	336.46 ± 46.00	307.60 ± 44.73	0.138
HDL-c (mg/dL)	22.79 ± 7.05	20.67 ± 9.04	19.57 ± 8.76	0.305
T-cho (mg/dL)	104.00 ± 27.30	100.33 ± 26.92	103.33 ± 27.40	0.901
Ca (mg/dL)	22.73 ± 1.40	25.75 ± 1.38	23.85 ± 1.55	0.160
Mg (mg/dL)	2.80 ± 0.18	3.28 ± 0.22	2.97 ± 0.20	0.264
IP (mg/dL)	7.30 ± 0.80	8.11 ± 0.78	7.49 ± 0.79	0.513
IgG (mg/mL)	7.43 ± 1.37	7.56 ± 1.62	9.69 ± 1.27	0.479
TNF-α (pg/mL)	148.78 ± 37.49	134.87 ± 54.60	134.94 ± 43.69	0.834

C diet: 3.0% fish meal and 0% black soldier fly larvae (BSFL) meal; L diet: 1.5% fish meal and 1.5% BSFL meal; H diet: 0% fish meal and 3.0% BSFL meal; ALP = Alkaline phosphatase; GOT = Glutamic oxaloacetic transaminase; GGT = Gamma glutamine transpeptidase; LDH = Lactate dehydrogenase; HDL-c = High-density lipoprotein cholesterol; T-cho = total cholesterol; IP, Inorganic phosphorus; IgG, Immunoglobulin G; TNF-α, Tumor necrosis factor-α; Data: mean ± SE; Control diet: *n* = 14; L diet: *n* = 13; H diet: *n* = 15.

**Table 4:** Maltase and Sucrase activities in jejunal and ileal mucosae of laying hens with different dietary treatments.

Disaccharidase Activities (mU/mg)	Diets			*p*-Value
	C Diet	L Diet	H Diet	
Jejunum				
Maltase activity	0.949 ± 0.060	0.845 ± 0.058	0.868 ± 0.079	0.971
Sucrase activity	0.678 ± 0.028	0.688 ± 0.033	0.687 ± 0.032	0.344
Ileum				
Maltase activity	0.397 ± 0.030 ^ab^	0.318 ± 0.026 ^b^	0.429 ± 0.032 ^a^	0.025
Sucrase activity	0.165 ± 0.042	0.111 ± 0.025	0.240 ± 0.078	0.218

C diet: 3.0% fish meal and 0% black soldier fly larvae (BSFL) meal; L diet: 1.5% fish meal and 1.5% BSFL meal; H diet: 0% fish meal and 3.0% BSFL meal; Data: mean ± SE; *n* = 14; Mean values within a row with different superscripts represent the statistical differences in multiple comparison (*p* < 0.05).

**Table 5:** Villus height, crypt depth, villi/crypt, and goblet cell density in the intestine of laying hens with different dietary treatments.

Intestines	Diets			*p*-Value
	C Diet	L Diet	H Diet	
Duodenum				
Villi height (µm)	1082.95 ± 51.08	1031.17 ± 51.81	1040.39 ± 26.87	0.803
Crypt depth (µm)	168.77 ± 5.96	174.62 ± 5.43	176.09 ± 7.14	0.681
Villi/Crypt	6.57 ± 0.44	5.95 ± 0.30	6.09 ± 0.35	0.462
Goblet cell density (10^3^/mm^2^)	2.21 ± 0.16	1.86 ± 0.07	1.78 ± 0.08	0.088
Jejunum				
Villi height (µm)	793.47 ± 53.03	771.34 ± 37.3	817.66 ± 44.70	0.773
Crypt depth (µm)	104.52 ± 4.80	113.1 ± 4.46	108.38 ± 4.59	0.320
Villi/Crypt	7.63 ± 0.44	6.89 ± 0.32	7.62 ± 0.43	0.337
Goblet cell density (10^3^/mm^2^)	2.02 ± 0.14	1.62 ± 0.12	1.61 ± 0.17	0.085
Ileum				
Villi height (µm)	529.44 ± 24.64	561.27 ± 31.87	545.65 ± 14.03	0.787
Crypt depth (µm)	87.45 ± 4.50	90.81 ± 3.96	93.49 ± 6.50	0.705
Villi/Crypt	6.14 ± 0.26	6.20 ± 0.25	6.08 ± 0.31	0.957
Goblet cell density (10^3^/mm^2^)	1.78 ± 0.14	1.65 ± 0.13	1.44 ± 0.06	0.139

C diet: 3.0% fish meal and 0% black soldier fly larvae (BSFL) meal; L diet: 1.5% fish meal and 1.5% BSFL meal; H diet: 0% fish meal and 3.0% BSFL meal; Data: mean ± SE; *n* = 15.

**Table 6:** The ten most abundant microbial pathways with significant differences in the caecum of laying hens with different dietary treatments.

Pathways	Pathway Enrichment Ratio (%)			*p*-Value
	C Diet	L Diet	H Diet	
β-glucosidase	2.37 ± 0.10 ^a^	2.23 ± 0.06 ^ab^	2.07 ± 0.09 ^bc^	0.041
Long-chain acyl-CoA synthetase	2.41 ± 0.10 ^a^	2.20 ± 0.06 ^ab^	2.06 ± 0.10 ^bc^	0.031
UDP glucose 6-dehydrogenase	2.36 ± 0.09 ^a^	2.25 ± 0.06 ^ab^	2.06 ± 0.09 ^bc^	0.041
periplasmic protein TonB	2.43 ± 0.13 ^a^	2.18 ± 0.08 ^ab^	2.05 ± 0.13 ^bc^	0.043
Bile acid: Na+ symporter, BASS family	2.41 ± 0.12 ^a^	2.20 ± 0.07 ^ab^	2.05 ± 0.11 ^bc^	0.041
K^+^-stimulated pyrophosphate-energized sodium pump	2.33 ± 0.08 ^a^	2.26 ± 0.05 ^ab^	2.08 ± 0.07 ^bc^	0.045
Carboxynorspermidine decarboxylase	2.34 ± 0.08 ^a^	2.25 ± 0.06 ^ab^	2.08 ± 0.07 ^bc^	0.034
Saccharopine dehydrogenase	2.33 ± 0.08 ^a^	2.25 ± 0.05 ^ab^	2.09 ± 0.07 ^bc^	0.050
Alpha-D-xyloside xylohydrolase	2.41 ± 0.09 ^a^	2.17 ± 0.07 ^ab^	2.09 ± 0.08 ^bc^	0.020
6-pyruvoyltetrahydropterin synthase	2.38 ± 0.10 ^a^	2.23 ± 0.21 ^ab^	2.06 ± 0.10 ^bc^	0.050

C diet: 3.0% fish meal and 0% black soldier fly larvae (BSFL) meal; L diet: 1.5% fish meal and 1.5% BSFL meal; H diet: 0% fish meal and 3.0% BSFL meal. Mean values within a row with different superscripts represent the statistical differences in multiple comparison (*p* < 0.05); Data: mean ± SE; *n* = 15.

## Data Availability

The raw data used in this study is available from the corresponding author on reasonable request.

## Supplementary Materials

The following supporting information can be downloaded at: https://www.mdpi.com/article/10.3390/ani13162629/s1, Table S1: Diet Ingredients and proximate nutrients; Table S2: The performance of laying hens with different dietary treatments; Table S3: The KEGG pathways of caecal microbiota from laying hens with different dietary treatments; Figure S1: The relative microbial abundance in the gut of laying hens with different diets at the family level.
