# Supplementary material for: Long-Term Dietary Fish Meal Substitution with the Black Soldier Fly Larval Meal Modifies the Caecal Microbiota and Microbial Pathway in Laying Hens
Source: Animals (Basel). 2023 Aug 15;13(16):2629. doi: 10.3390/ani13162629 (PMC10451910; doi:10.3390/ani13162629)
Supplement: Supplementary file 1 [file animals-13-02629-s001.zip › Supplementary Figure S1.pdf]

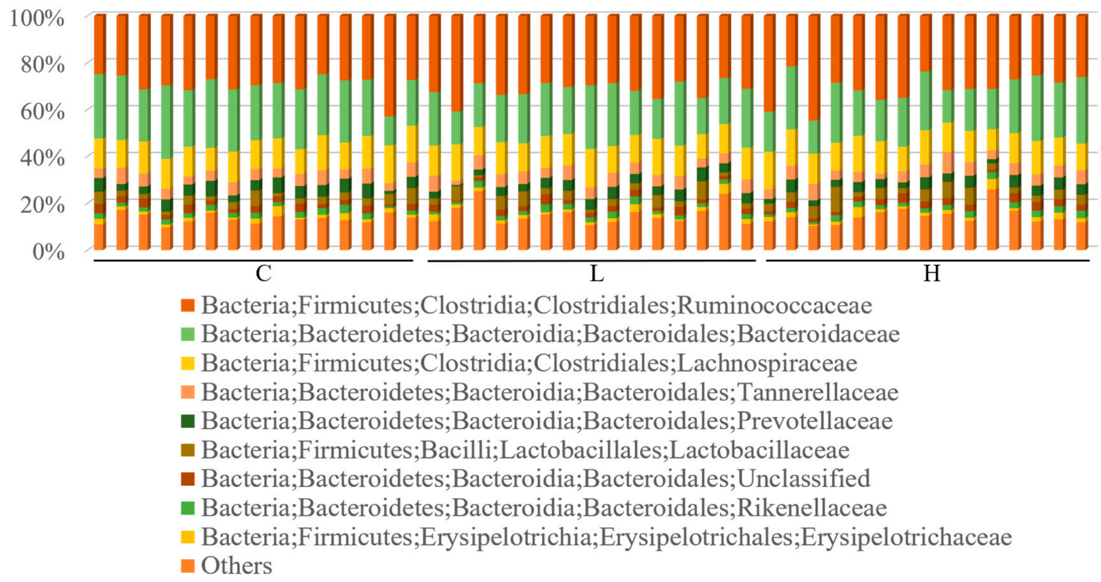

Figure S1. The relative microbial abundance in the gut of laying hens with different diets at the family level. C diet: 3.0% fish meal and 0% black soldier fly larvae (BSFL) meal; L diet: 1.5% fish meal and 1.5% BSFL meal; H diet: 0% fish meal and 3.0% BSFL meal.
