# Supplementary material for: Long-Term Dietary Fish Meal Substitution with the Black Soldier Fly Larval Meal Modifies the Caecal Microbiota and Microbial Pathway in Laying Hens
Source: Animals (Basel). 2023 Aug 15;13(16):2629. doi: 10.3390/ani13162629 (PMC10451910; doi:10.3390/ani13162629)
Supplement: Supplementary file 1 [file animals-13-02629-s001.zip › Supplementary Table S1.pdf]

Table S1. Diet Ingredients and proximate nutrients [1]

Ingredients (% in fresh matter)	Control	1.5% BSFL diet	3% BSFL diet
Maize grain	59.0	59.0	59.0
Soybean meal	21.0	21.0	21.0
Calcium carbonate	8.0	8.0	8.0
Rice bran	4.0	4.0	4.0
Fish meal	3.0	1.5	0.0
BSFL meal	0.0	1.5	3.0
Corn oil	2.0	2.0	2.0
Salt	1.0	1.0	1.0
Tricalcium phosphate	1.0	1.0	1.0
Vitamin and mineral mix	1.0	1.0	1.0
Proximate nutrients (% in dry matter) and energy content			
Dry matter [2]	88.0	88.0	88.0
Crude protein [2]	19.1	19.1	19.8
Crude fat [2]	6.2	6.2	6.5
Crude fiber [2]	3.7	3.4	3.4
Crude ash [2]	12.4	12.2	12.6
NFE [3]	46.6	47.0	45.7
ME (Mcal/kg) [3]	3.27	3.29	3.29

BSFL = Black Soldier Fly Larvae; NFE = Nitrogen Free Extract; ME = Metabolizable Energy.

[1] Note. Data from "Egg quality and laying performance of Julia laying hens fed with black soldier fly (*Hermetia illucens*) larvae meal as a long-term substitute for fish meal" by Zhao *et al.*, 2022, *Poultry science* 101, 101986. Copyright (2022) by Elsevier Inc.

[2] Analyzed data.

[3] Calculated data.
