# Supplementary material for: Long-Term Dietary Fish Meal Substitution with the Black Soldier Fly Larval Meal Modifies the Caecal Microbiota and Microbial Pathway in Laying Hens
Source: Animals (Basel). 2023 Aug 15;13(16):2629. doi: 10.3390/ani13162629 (PMC10451910; doi:10.3390/ani13162629)
Supplement: Supplementary file 1 [file animals-13-02629-s001.zip › Supplementary Table S2.pdf]

Table S2. The performance of laying hens with different dietary treatments [1]

Item	Diets		
	Control diet	1.5% BSFL diet	3.0% BSFL diet
Body weight (g) [2]	1455.79 ± 117.287 [a]	1545.03 ± 176.932 [ab]	1573.86 ± 133.878 [b]
Feed intake (g) [2]	133.48 ± 4.955	134.59 ± 3.969	137.28 ± 1.661
Laying rate (%) [2]	83.84 ± 5.547	85.61 ± 11.261	83.84 ± 5.647
Egg weight (g) [3]	56.59 ± 0.363 [b]	58.20 ± 0.263 [a]	59.16 ± 0.679 [a]
Mortality (%) [4]	17.50 ± 6.083	16.50 ± 9.330	10.44 ± 1.166

[1.] Note. Data from "Egg quality and laying performance of Julia laying hens fed with black soldier fly (*Hermetia illucens*) larvae meal as a long-term substitute for fish meal" by Zhao *et al.*, 2022, *Poultry science* 101, 101986. Copyright (2022) by Elsevier Inc.

[2.] The analysis was performed with the mean of the performance of all individuals in each group over 4 weeks; Control diet: n = 24; 1.5% BSFL meal diet: n = 24; 3.0% BSFL meal diet: n = 26.

[3.] The analysis was performed with the mean of the twelve eggs that close to the average value of all eggs weight in each group, n = 12.

[4.] The analysis was performed with three replicates in each dietary treatment.

Data: mean ± SD; Mean values within a row with different superscripts letters represent the statistical differences ($P < 0.05$).
